# Occupational Mercury Exposure at a Fluorescent Lamp Recycling Facility — Wisconsin, 2017

**DOI:** 10.15585/mmwr.mm6727a3

**Published:** 2018-07-13

**Authors:** Erica Wilson, Jeffery S. Lafferty, Robert Thiboldeaux, Carrie Tomasallo, Barbara Grajewski, Ryan Wozniak, Jonathan Meiman

**Affiliations:** ^1^Epidemic Intelligence Service, CDC; ^2^Bureau of Environmental and Occupational Health, Wisconsin Department of Health Services; ^3^Public Health Madison & Dane County, Madison, Wisconsin.

On May 9, 2017, Public Health Madison & Dane County contacted the Wisconsin Division of Public Health for assistance with investigation of mercury exposure among workers at a fluorescent lamp recycling facility. Public Health Madison & Dane County had been contacted by the Wisconsin Department of Natural Resources as part of an investigation of potential environmental contamination at the facility. Fluorescent lamps are composed of a phosphor-coated glass tube containing mercury vapor and argon. During the recycling process, lamps are crushed, releasing mercury vapor and mercury-containing dusts. State and county health officials, in collaboration with Wisconsin Department of Natural Resources, conducted an investigation of mercury exposure of workers and an environmental assessment of the facility, surrounding areas, and worker vehicles. All five workers who were tested had urine mercury levels exceeding the American Conference of Governmental Industrial Hygienists (ACGIH) biologic exposure index of 20.0 *μ*g/g creatinine, and two had tremor on physical exam. Workers wore inadequate personal protective equipment (PPE). Mercury levels in indoor air varied within the building, with a maximum of 207.4 *μ*g/m^3^ at floor level on the crushing platform, approximately eightfold higher than the ACGIH threshold limit value of 25 *μ*g/m^3^ (*1*). Mercury also was found in workers’ vehicles, indicating risk for take-home exposure. Workers at risk for mercury exposure need to have access to and consistently wear National Institute of Occupational Safety and Health (NIOSH)-approved respiratory protection for mercury vapor, nitrile or other suitable gloves to prevent contact exposure, and disposable suits with booties and change shoes before leaving the worksite to prevent take-home exposures.

On May 12, 2017, the Wisconsin Division of Public Health, Public Health Madison & Dane County, and the Occupational Safety and Health Administration (OSHA) conducted a facility site visit to assess the work environment, interview workers, and perform environmental monitoring. Workers were advised to be tested for mercury exposure, and spot urine testing was offered at the time of the site visit. A case of mercury exposure was defined as a urine spot mercury level above the ACGIH biologic exposure index of 20.0 *μ*g/g creatinine in a facility worker. Twenty-four–hour urine samples were not obtained because of potential contamination at the work site during urine collection. Workers who received a diagnosis of mercury exposure were referred to an occupational health clinic for further evaluation. All workers were asked to participate in a survey that included employment history, symptoms of mercury toxicity, PPE use, and medical and social history.

The 6,000–square-foot lamp recycling facility consisted of a large storage area with offices and kitchenette at the front and a break room at the back. A processing area with a drum-type crusher was located on one side of the storage area, and a bay door opened from the outside into the storage area on the opposite side. Ambient air sampling of the facility was conducted using a Lumex RA-915+ mercury vapor analyzer (Ohio Lumex Co., Inc.). Because of the timing of the unannounced visit, sampling was conducted when the facility was not processing; the bay door was open during sampling. Mercury vapors were measured just above floor level to assess spilled mercury and phosphor powder and at breathing height (approximately 4–5 feet above floor level) to assess worker exposure levels. The processing platform was approximately 4-feet high. All areas of the facility were sampled, including the facility entrance, reception area, office, kitchenette, hallways, bathroom, lockers, break room, and processing floor.

Potential for take-home mercury exposure was assessed by wipe-sampling workers’ vehicle foot pedals on June 20. All workers declined assessment of their homes for mercury contamination. Wisconsin Department of Natural Resources sampled water and fish from two nearby ponds on May 25 and June 19 to evaluate potential contamination from the facility.

Seven persons worked at the facility, including the owner-manager and six persons who worked in processing, administration, or as drivers. Workers’ mean age was 35 years (range = 23–50 years), six of seven workers were male, and mean duration of employment at the facility was 2 years (range = 0–5 years). Five workers had worked at the facility for a previous owner who had been cited by OSHA for elevated air mercury levels and failure to use respirators after an investigation on September 2, 2016. Appropriate respirators with mercury vapor cartridges were provided to workers after that investigation.

Spot urine samples were obtained from four of the seven workers; a fifth worker’s spot urine sample was obtained 1 week later. Two workers declined testing. All five tested workers met the case definition for mercury exposure; the average urine mercury/creatinine ratio was 49.6 *μ*g/g creatinine (range: >23.8–71.2 *μ*g/g creatinine). Follow-up during June–September 2017 for three workers evaluated at an occupational health clinic and one evaluated at a primary care clinic included repeat spot urines and 24-hour urine collections (Table 1). Repeat testing showed a decrease in mercury levels in urine, blood, or both for two workers and indeterminate results in one worker. One worker continued to have elevated blood and urine mercury levels indicative of continued exposure.

**TABLE 1:** Urine and blood mercury test results and personal protective equipment usage for workers at a fluorescent lamp recycling facility — Wisconsin, 2017

Worker ID*	Years at facility	Duties	Test 1 (0 wks)^†^	Test 2 (2–3 wks)			Test 3 (8–10 wks)		Test 4 (11 wks)	Test 5 (15 wks)	Use of PPE
			Spot urine (*μ*g/g Cr)^§^	Blood (*μ*g/L)^¶^	Spot urine (*μ*g/g Cr)^§^	24-hr urine (*μ*g/L)**	24-hr urine (*μ*g/L)**	Blood (*μ*g/L)^¶^	24-hr urine (*μ*g/L)**	24-hr urine (*μ*g/L)**	
1	0	Management	—^††^	—^††^	—^††^	—^††^	—^††^	—^††^	—^††^	—^††^	Unknown
2	4	Sales/Logistics/Admin	71.2	24	>75	—^††^	44	38	—^††^	—^††^	No
3	1.5	Driver	39.2	—^††^	—^††^	—^††^	—^††^	—^††^	—^††^	—^††^	Unknown
4	2	Warehouse/Sorting/Processing	64.0	—^††^	—^††^	37	—^††^	—^††^	—^††^	—^††^	Inconsistent
5	5	Warehouse/Sorting/Processing	>23.8	—^††^	—^††^	28	—^††^	—^††^	—^††^	—^††^	No
6	1.5	Warehouse/Sorting/Processing	50.0	12	81.4	—^††^	85	35	86	109	Inconsistent
7	0	Driver	—^††^	—^††^	—^††^	—^††^	—^††^	—^††^	—^††^	—^††^	Unknown

**Abbreviation:** Cr = creatinine, PPE = personal protective equipment.

* Workers 1 and 7 declined testing.

^†^ First test May 2017.

^§^ American Conference of Governmental Industrial Hygienists biologic exposure index = 20.0 *μ*g/g Cr.

^¶^ American Conference of Governmental Industrial Hygienists biologic exposure index = 15 *μ*g/L.

** The biologic exposure index is determined by the American Conference of Governmental Industrial Hygienists as a guideline to assist in the control of health hazards by industrial hygienists; however, no biologic exposure index or consensus standard exists for 24-hour urine testing. The analyzing lab indicated that the normal range is <10 µg/L.

^††^ Not tested.

Four workers completed the survey. The symptom most commonly reported was breathing difficulty (reported by all four workers), followed by memory loss, irritability, insomnia, headaches, and weakness (three of four). No worker reported difficulty walking. One worker reported tremor, and another reported muscle twitches.

Two of three processing workers wore rubber gloves, respirators, goggles, and disposable coveralls only while processing; the third wore only cloth gloves. Only one worker wore booties. One worker said he only started wearing PPE within the past month. No workers changed clothes or shoes before leaving the facility. Three workers attended an occupational health appointment with a physician during the 5 months after the initial investigation. One patient had no physical findings of mercury toxicity, one had tremor of the hands and head, and one had tremor of the fingers and a Mini Mental Status Exam score of 27/30 (normal >24/30). No prior Mini Mental Status Exam score was available for comparison.

Spot air sampling found mercury vapor concentrations of 0.2–6.8 *μ*g/m^3^ outside of processing areas, with differences of up to 1 *μ*g/m^3^ between ground level and breathing height measurements; higher mercury levels at the ground were reported, compared with breathing height (Table 2). Inside the processing area, mercury levels were 9.0 *μ*g/m^3^ at the entrance and reached a maximum of 207.4 *μ*g/m^3^ on the floor of the processing platform and 99.7 *μ*g/m^3^ at breathing level on the processing platform ramp.

**TABLE 2:** Mercury vapor air sampling results at a fluorescent lamp recycling facility* — Wisconsin, 2017

Location	Mercury vapor concentration (*μ*g/m^3^)^†^	
	Floor level	Breathing height^§^
20 Feet from warehouse entrance	—^¶^	0.3
Warehouse entrance	—^¶^	0.2–1.7
10 Feet inside warehouse	—^¶^	3.2–3.5
20 Feet inside warehouse, ground level	5.7–6.0	—^¶^
Entrance of warehouse office	—^¶^	4.0–5.0
Inside office	4–4.5	2.5–3.0
Reception area	3.5	3.8
Main office	3.7	2.7
Main office kitchenette	—^¶^	3.1–3.5
Hallway	5.5	4.8
Office bathroom	—^¶^	5.4
Break room	—^¶^	6.4–6.8
Center of warehouse	—^¶^	3.1
Near lockers and Tyvek suits	—^¶^	2.7–3.0
Back storage area, near forklift	—^¶^	2.8
Entrance to processing area	—^¶^	9
10 Feet inside processing area, near crushing door	—^¶^	38.1–57.9
On top of crushing platform	138.5–207.4	32.9
Back of processing, side door	—^¶^	82.8
Processing floor	85.1–100	—^¶^
Processing ramp	—^¶^	99.7

* Using a Lumex RA-915+ mercury vapor analyzer (LumexCo. Inc.).

^†^ Occupational Safety and Health Administration permissible exposure limit = 0.1 *μ*g/m^3^; National Institute for Occupational Safety and Health recommended exposure limit = 0.05 *μ*g/m^3^; and American Conference of Governmental Industrial Hygienists threshold limit value = 0.025 *μ*g/m^3^.

^§^ Breathing height is approximately 4–5 feet above the floor.

^¶^ Not tested.

Wipe samples from the cars of two workers determined the presence of mercury, indicating a risk for take-home exposure. Samples of water and fish from two nearby ponds found mercury levels consistent with regional freshwater mercury levels.

## Discussion

Workers at the lamp recycling facility were exposed to mercury in the air, had elevated urine mercury levels, and experienced signs and symptoms of mercury toxicity. Previous investigations have reported that 33% of mercury is released from bulbs in the first 8 hours after breakage (*2*), and that processing in an open area decreases exposure (*3*). According to a U.S. Department of Energy report, approximately 3.8 billion fluorescent lamps were installed in the United States during 2010 (*4*). Recycling used fluorescent lamps prevents release of mercury and other metals into the environment and allows reclamation of materials for reuse. Wisconsin state law requires businesses and institutions to recycle used fluorescent bulbs (*5*).

The risk for mercury exposure in the manufacturing of fluorescent lamps has been known since the first investigation of a fluorescent lamp manufacturer in 1965 reported elevated urine mercury levels among glass blowers who made and repaired lamps (*6*). However, risks associated with fluorescent lamp recycling have not been well documented. A case study reported membranous nephropathy and elevated mercury levels in two workers at a fluorescent lamp recycling facility (*7*), and two studies have demonstrated levels of mercury vapor exceeding OSHA permissible exposure limit during processing of fluorescent lamps using drum-type crushers (*3*,*8*).

In this investigation, environmental measurements likely underestimated workers’ exposure to mercury because processing was suspended during the site visit and the bay door was open during sampling. Although the spot environmental mercury vapor concentrations measured in this investigation cannot be directly compared with the time-weighted averages used in OSHA (*9*), NIOSH (*10*), and ACGIH (*1*) guidelines, this investigation indicates increased risk for adverse health effects from mercury exposure to workers in fluorescent lamps recycling facilities, with potential for take-home exposure and environmental contamination. Despite changes implemented after the 2016 OSHA investigation that included access to correct respirators, workers did not consistently use PPE and had elevated mercury levels. To mitigate risks to workers, employers need to implement engineering control technology and housekeeping (mercury appropriate vacuum, regular cleaning of surfaces with correct disposal of cleaning equipment) to reduce mercury contamination at their facilities. A clear protection program policy needs to be provided, and workers need to receive training in PPE and wear the PPE needed for their task. In addition to reducing mercury exposure with engineering and administrative controls, regular mercury control housekeeping needs to be used. Periodic monitoring can be considered to ensure employee exposures remain within existing recommended limits.

**Summary**

**What is already known about this topic?**

The risk for mercury exposure from manufacture of fluorescent lights has been known for many years; risks for exposure from recycling are not well documented.

**What is added by this report?**

An investigation of environmental contamination at a fluorescent light recycling facility in Wisconsin in 2017 found elevated mercury levels among five of seven workers and clinical signs of mercury toxicity in two. Use of personal protective equipment was inconsistent, and mercury levels for inside air exceeded recommended thresholds.

**What are the implications for public health practice?**

Employers at fluorescent light recycling facilities need to implement control technology, housekeeping, and exposure monitoring, and provide recommended PPE and training to their workers to reduce mercury exposures at their facilities.
